# Always to Your Interest to Read These Selections

**Published:** 1897-10

**Authors:** 


### ALWAYS TO YOUR INTEREST TO READ THESE SELECTIONS.

The Department of Agriculture will inaugurate a study of the needs of foreign countries as to the type and quality of horses required, with the hope that our horsebreeders may turn their attention to the production of such horses as will be assured a foreign market. The consular and diplomatic departments will be made the medium for obtaining such information. The purchase of horses for foreign markets the past two years has been such as to establish the conclusions that our facilities for raising are superior to any country in the world, and, when the type and character of the horse needed is better known, it should not be difficult for us to obtain this market.

Prof. Cossart Ewart has succeeded in obtaining another hybrid by the same zebra sire. This one is from an Irish cob mare, bay, with black points, 14.1 hands high. It was twelve days beyond the usual time of a mare. The hybrid colt is much lighter in color than the first one; the body of a reddish tinge or light bay, and the stripes red-brown, not so strongly black as the first hybrid. The first mare used in this telegonic experiment was subsequently bred to an Arab horse, and is early due to foal. Much interest is wrapped up in the birth of this foal.

A recent sale at Belfast, Ireland, of horses, all choice selections, taken there from Kentucky, ranged for the twenty-nine sold from $204 to $817. It is said that over two thousand prospective buyers attended this sale.

Grass-bred and fed Western bronchos recently completed a trip of 2400 miles under the saddle in less than three months. They are said to have arrived in good condition. Surely no better evidence of endurance could be asked for.

The medical societies of New York State are taking such action as will abate the free dispensary nuisance. The aid of the Legislature will be invoked. This step in the Empire State will be followed in other States, for it is growing more difficult every year for young men to enter practice, though better equipped than ever before. It is a wrong for a State Legislature to appropriate moneys for the great proportion of these institutions that only exist by favor of the public treasury—not by public demand.

A loss in a racing greyhound bitch was recently decided in the English courts. The expert testimony was on the existence of certain pathological adhesions in the chest, as to the length of time said adhesions had existed.

Strychnia is said to have been discovered in an analysis of portions of food given to a promising two-year-old in the $7500 colt race at Goshen, N. Y., in September. Hanging would be too good for the one committing such a crime.

Glasgow, Scotland, held a public meeting in September to discuss measures and means of stamping out glanders in that city. Without proper restraint or control it seems that there have been dispersal sales in that city of large numbers of horses from infected stables which for years had not been free from this serious disease. Surely there should be an awakening of public sentiment to the need of district inspectors in all large cities to save the great monetary losses incurred, not to mention the protection to man from this loathsome disease.

"Old Nig," a famous bus-horse of Addison, N. Y., died March 31, 1890, at the age of forty years. He made 83,760 trips between the station and the hotel, and is estimated to have consumed sixty-two tons of hay and 8307 bushels of oats.

A LIBRARY SECURED THROUGH A BALKY HORSE.—While Ex-Governor Flower, Ex-Governor Cornell, and Hon. Joseph Hendrix were driving over the Cornell campus, near the veterinary department, one of the horses suddenly became balky and refused to go, although repeatedly urged to do so by the whip of the driver. The party, therefore, went into the building to await the submission of the horse, and while there met Prof. James Law. The library of the department only contains fifteen volumes, and Mr. Cornell suggested to Ex-Goverenor Flower that he contribute enough to increase the number to a respectable amount. Mr. Flower turned to Dr. Law and asked him what amount would be needed, to which the doctor replied $5000. To the infinite surprise of those present Mr. Flower drew out his check-book and made out a check for the requisite amount. He then returned to the carriage with his companions, and the horse, having come to a proper frame of mind during their absence, started off without any further protest.

The more thorough the light shed upon the serum-treatment of hog-cholera, the more promising its results become, and the greater the hopes entertained that it will prove a solution of this great scientific question to the veterinarians and monetary burden to our stock-raisers.

